# Increase in landslide activity after a low-magnitude earthquake as inferred from DInSAR interferometry

**DOI:** 10.1038/s41598-022-06508-w

**Published:** 2022-02-17

**Authors:** S. Martino, M. Fiorucci, G. M. Marmoni, L. Casaburi, B. Antonielli, P. Mazzanti

**Affiliations:** 1 Earth Sciences Department of “Sapienza” University of Rome and CERI - Research Centre for Geological Risks, P.le Aldo Moro n.5, 00185 Rome, Italy; 2 NHAZCA S.R.L., Spin-Off Company of “Sapienza” University of Rome, Via Vittorio Bachelet n.12, 00185 Rome, Italy

**Keywords:** Environmental sciences, Natural hazards

## Abstract

On August 16th, 2018, a Mw 5.1 earthquake struck the Molise region (central Italy), inducing 84 earthquake-triggered landslides that predominantly involved soil covers of clayey materials and flysch on gently dipping slopes. To quantify the spatiotemporal landslide activity in the months immediately after the earthquake, a differential SAR interferometry (DInSAR) analysis was performed for a time span from 2 years before to one year after the earthquake, recognising both first-time and reactivated landslides. The results showed a clear increase in landslide activity following the low-magnitude earthquake with respect to the activities recorded in the same months of the previous years. Several coherent landslides (earth slides and earth flows) were observed following seasonally recurrent rainfall events. Such increases were observed for both reactivated and first-time landslides, showing decreases in inactive periods and activity over longer periods. Furthermore, the spatial density distribution of the landslides was investigated in the postseismic time interval along transects perpendicular and parallel to the direction of the tectonic element responsible for the seismic event. An asymmetrical distribution was deduced parallel to the fault strike with a higher number of landslides located inside the compressional sector according to a strike-slip faulting mechanism.

## Introduction

Earthquake-triggered landslides (EqTLs) represent one of the major geological hazards in mountainous and hilly areas and are one of the main seismically induced ground effects^1^. Under particular conditions, postseismic ground effects are documented to be as effective as coseismic effects^2^, inducing or promoting prolonged erosion and perturbations of stream network muss budgets by increasing the sediment load in rivers^3–5^. These conditions pertain to natural slopes in tectonically active areas, where seismic shaking can weaken rock masses or soils, leaving them free to be flushed and eroded from hillslopes^3^.

Most of the induced ground effects that contribute to these enhanced rates are represented by disrupted and coherent landslides that involve damaged rock masses or loosened soils and regolith^6^, debris flows or reactivation of pre-existing or coseismic landslides^1,7–9^.

Impulsive shaking plays a prominent role in increasing the landscape sensitivity of the involved areas to meteoclimatic forcing after earthquakes^7^, which can be quantified by a reduction in critical rainfall threshold^1,10–12^ or a reduction in strength that reflects an increase in landslide rates. This erosive response is proved to be linked to the character of seismic shaking^3^ (i.e., usually expressed by the peak ground acceleration (PGA) or the Arias intensity) and is markedly effective in the epicentral areas, where most EqTLs are registered^13,14^ and where the most effective tectonic and seismological control in the near-fault region is expected^15–17^. The increased disequilibrium after earthquakes results in a fast transient pulse and is recovered over years depending on the earthquake moment magnitude^1,7^.

While increases in erosion and landslide rates were registered after strong earthquakes in recent decades (e.g., the 2008 Mw 7.9 Wenchuan, 2005 Mw 7.6 Kashmir, and 1999 Mw 7.7 Chi Chi earthquakes) on predisposed slopes featuring a weak soil cover or weakened substratum and high susceptibility to landslides, this effect could be equally relevant after relatively low-magnitude earthquakes, contributing as an acquired legacy to the aggravation of postseismic rainfall-induced landslide susceptibility^18^.

Control by tectonic and faulting mechanisms in the distribution of EqTLs in coseismic stages, reported in datasets of ground failures worldwide^1,19^, has also been evaluated postseismic stages, analysing the spatial density and directional distributions with respect to the strike-slip faulting mechanism.

Satellite SAR interferometry has been largely used for updating landslide inventories, assessing the state of activity by means of multitemporal techniques such as advanced differential synthetic aperture radar interferometry (DInSAR)^20–22^ with integration of in situ and thematic data. The multitemporal approach involves large time spans through the stacking of tens to hundreds of SAR images. Additionally, the conventional single-pair DInSAR technique has been employed to monitor landslide displacements^23–25^.

For this type of study, DInSAR analysis allows the detection of (1) fast movements derived from a single interferogram analysis^23^, which allows measurement of deformation that occurs in a few days (6 days as the minimum temporal baseline for the Sentinel-1 sensor), and (2) impulsive phenomena such as landslide reactivations and nonlinear movements such as the acceleration of a landslide after a rainy period.

The results of a 3 year-long interferometric analysis aimed at inventorying landslide reactivations after an earthquake are presented here. The case study is represented by the epicentral areas of the August 16th, 2018, Mw 5.1 Montecilfone earthquake, where 84 EqTLs were surveyed, highlighting the relevance of rainfall in aggravating the coseismic scenario. Due to the low earthquake magnitude and the EqTLs detected in the case study, the resulting scenario seems to be overestimated, suggesting a combined action of preparatory and triggering factors^26^.

## Geological and geomorphological framework

The study area struck by the Mw 5.1 Montecilfone earthquake is approximately 1000 km^2^ and is located on the NE side of the Molise region in a portion between the Frentani Mts. and Adriatic coast, representing the external part of the Apennine chain.

This region is characterised by a NE-verging fold-and-thrust belt revealed by the stacking of the most external tectonic units of the southern sector of the chain, locally covered by thrust-top deposits and a late Miocene-Pliocene terrigenous succession^27–29^. This geological setting was derived from the early Miocene to Pleistocene uplift of the chain, which led to the current regional morphostructural setting^28^.

More specifically, the sector of the Apennines under investigation is the result of three important tectonic phases^30^, which involved both surficial and deeper tectonic units derived from the deformation of the buried Apulian domain: (1) a compressive phase (lower Miocene—upper Pliocene), characterised by the development of E-vergent surficial thrusts; (2) a transcurrent phase (upper Pliocene—lower Pleistocene), characterised by the presence of N–S and E–W faulting; and (3) an extensional phase (middle Pleistocene—Holocene), characterised by direct SW–NE and NW–SE fault systems that are still active in the study area, as suggested by seismicity and landslide activity.

The study area presents a thick marine sequence of chain domains (Cenozoic) belonging to the Lagonegrese-Molisan Basin^27,28^; the deposits are mainly composed of scaly clays followed by arenaceous-marly and calcareous-marly flysches^27^, evaporites and chaotic terrains^27,28^ while thick siliciclastic sequences (Plio-Pleistocene) crop out in the foredeep. Since the middle Pleistocene, the study area has been affected by moderate tectonic uplift^31,32^ resulting in the formation of regressive sequences and terraced marine deposits that crop out in the hilly relief of Guglionesi, Petacciato and San Giacomo degli Schiavoni villages^33^. The successions of both domains (i.e., chain and foredeep) are covered by continental, marine and transitional deposits (Quaternary) that crop out widely in alluvial areas and close to the Adriatic coast, where their thickness reaches tens of metres^27,34,35^.

**Figure 1:**
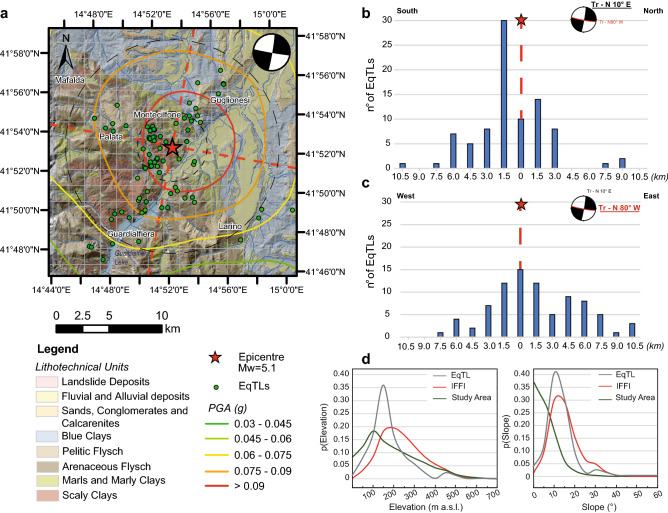
(**a**) Distribution of earthquake-triggered landslides (EqTLs) in the study area with respect to outcrops of lithotechnical units and PGA of the August 16th, 2018, mainshock. Focal mechanism and the traces of fault-plane solutions according to this mechanism are shown. (**b**,**c**) Distribution of EqTLs perpendicular to the fault strike (transect oriented N10°E) and parallel to the fault strike (transect oriented N80°W), respectively, directly surveyed in the field. (**d**) Frequency distribution of elevation (left) and slope (right) for 2018 EQtL (grey curve) with respect to both the total landslides (red curve) available in the generic IFFI landslide catalogue and the morphology of slopes in the entire study area (green curve). Map is created by ArcGis Desktop 10.8 (Licensee Customer Number: 274576—Sapienza University) using a hillshade model from the open source Shuttle Radar Topography Mission (SRTM) 1 Arc-Second Global DEM (https://earthexplorer.usgs.gov/).

The lithotechnical units that crop out in the study area can be described as follows^27^ (Fig. 1):

- Quaternary deposits: composed of (1) landslides and (2) terraced alluvial deposits of both continental and marine environments found near the river network and close to the Adriatic coast, respectively;
- Foredeep domain: composed of (1) blue clays that pass upwards to sands and conglomerates and ii) marine conglomerate-sandy deposits (Plio-Pleistocene);
- Chain domain: composed of (1) pelitic flysch belonging to the Vallone Ferrato Fm (Messinian—Tortonian), mainly composed of marls and clayey marls interlayered with sandstones and siltstones; (2) arenaceous flysch belonging to the Faeto Fm (Tortonian-Serravallian); and (3) scaly clays (lower Miocene—Upper Cretaceous) that form a tectonic melange consisting of clays and marly clays, with typical colouring from green to reddish, with a chaotic structure and micritic intercalations;
- Top-thrust basin transitional domain: composed of (1) calcarenites belonging to the Palombaro, Casalanguida and Larino Fms (middle Pliocene—lower Pliocene), (2) marly clays (middle Pliocene—lower Pliocene).

The lithologies that crop out justify a hilly relief with elevations up to 650 m a.s.l. and river valleys of variable extent. The central sector of the study area belongs to the lower basin of the Biferno River, while northern and southern sectors bound the basins of the Trigno and Fortore rivers.

The geotechnical features of the outcropping units make the Molise region one of the most landslide-prone areas in central and southern Italy^36^. In fact, it is typified by the presence of more than 28,000 landslides of different sizes and types of movement that cover an area of approximately 4461 km^2^ ^37–40^. In the epicentral area of the 2018 earthquake, approximately 7100 landslides were recognised over time, with sizes ranging from 100 m^2^ to 3 km^2^. Regarding the state of activity, dormant landslides account for approximately 72%, while active or stabilised landslides represent approximately 24% and 4%, respectively. These landslides show low to very low velocities and can be classified as flows, slides or complex landslides, according to Hungr et al.^41^. Moreover, very slow viscous deformation of the soil cover, such as soil creep and solifluction, can affect gently dipping slopes. Landslides are often driven by the presence of tectonic alignments or by fluvial networks and by surface erosion of runoff waters. Gravity-induced deformation mainly involves Quaternary deposits and the shallow and weathered portions of the bedrock. Deep phenomena involve the thick sequences of Plio-Pleistocene clays, especially in coastal areas^42–44^.

### Seismic and rainfall events on August 16th, 2018

The study area was struck by a Mw 5.1 earthquake that occurred on August 16th, 2018, at 18:19:04 (UTC) with an epicentre in Montecilfone (CB—Molise region—central Italy) (LAT 41.87; LONG 14.86, hypocentral depth 20 km); this event represented the mainshock of a longer seismic sequence, which started on April 25, 2018, and ended on September 4, 2018, and was followed by almost 840 low-magnitude earthquakes^45^ (Mw < 2.0).

The 2018 Montecilfone mainshock resulted in a low PGA of up to 0.12 g (http://shakemap.rm.ingv.it). Macroseismic surveying was conducted by the INGV QUEST Group^46^. In general, despite a low-damage scenario with respect to buildings in Montecilfone, Acquaviva Collecroce and Castelmauro municipalities (grade V-VI of EMS98 scale), a higher intensity level (up to VII) can be attributed if earthquake-induced ground effects are considered in accordance with the Environmental Seismic Intensity (ESI) scale^47^. The distribution of the epicentres appears to be slightly elongated in the E–W direction, similar to the epicentres of the 2002 San Giuliano di Puglia seismic sequence, located approximately 20 km south of the Montecilfone epicentral area. The focal mechanism of the 2018 mainshock indicates a dextral strike-slip rupture^45^, which is consistent with that elaborated for the 2002 earthquake and is identified in the Individual Seismogenic Source code as ITIS052 (San Giuliano di Puglia) and in the Composite Seismogenic Source code as ITCS003 (Ripabottoni-San Severo) by the Database of Individual Seismogenic Sources v. 3.2.1 (DISS v. 3.2.1- http://diss.rm.ingv.it/diss/). Valensise et al.^48^ added more information for the 2002 San Giuliano di Puglia earthquake, considering it due to a composite rupture of a 20 km-long segment of an important E–W-trending pre-existing subvertical fault. This event was located on the westward prolongation of the Mattinata fault zone, approximately 15 km west of the presumed western end of the Mw 6.7 rupture of July 30th, 1627 (corresponding to ITIS054: San Severo of the DISS; Terremoto della Capitanata), 30 km east of the Apennines axis. Valensise et al.^48^ also suggest that the entire system is active but that it is segmented up into 10- to 25 km-long portions. Given the relatively low magnitude of the event, the seismogenic source is not included in the DISS catalogue. Notwithstanding, the 2002 and 2018 Molise seismic sequences highlight the occurrence of active faulting in this area, where relevant earthquakes have not previously been recorded. This zone is located approximately 50 km east of the extensional Appenninic belt and approximately 50 km west of the dextral strike-slip Mattinata fault^49^.

During the three days before the seismic event, the epicentral area also experienced intense cumulative rainfall ranging from 120 to 150 mm, as recorded by the rain gauges closest to the epicentre (Ponte Liscione and Palata weather stations, 4.5 km S and 6 km NNE from the epicentre, respectively). To eliminate the action of rain alone as the main triggering factor of the surveyed landslides, a statistical analysis of maximum daily and hourly rainfall intensity data was performed by Martino et al.^26^ and indicated that the August 2018 rainfall event cannot be classified as exceptional with respect to other rainfall events that occurred in the same area during recent decades (see supplementary materials).

The EqTLs resulted from the coupled action of simultaneous preparatory and triggering factors characterised by different hazards. The longer-term factor is referred to as the seismic trigger, which has a return period ranging from 150 to 200 years. The shorter-term factor can be attributed to rainfall triggers, characterised by maximum return periods of 17 and 13 years if time windows of 1 h and 3 days are considered, respectively. It follows that the contribution of the earthquake, although prepared by rainfall, appears to be a necessary condition to justify the landslide scenario surveyed immediately after the August 2018 mainshock, implying a coseismic scenario.

### Landslide scenario induced by the August 16th, 2018, earthquake

In the days immediately after the 2018 Montecilfone earthquake, a detailed field survey of earthquake-induced ground effects was performed by the Sapienza fast-response team^26^, inventorying 84 earthquake-triggered landslides (EqTLs)^50^ and 4 coseismic ground cracks.

The observed effects were surveyed and geolocalised in the field following the standardised procedure reported in Martino et al.^51^; this procedure focused on the distinction of clear clues for landslide activation (or reactivation). In this way, all surveyed landslides were compared with the available landslide catalogue (IFFI, Italian Landslide Inventory, available online at https://idrogeo.isprambiente.it/app/) to assess the number of landslide reactivations with respect to first occurrences^52^ in the coseismic interval; this analysis revealed that 37.5% of EqTLs were reactivated pre-existing landslides. The prevalent landslide mechanisms included earth slides and earth flows, which involved slopes with inclinations ranging from 10° to 15° according to lithological unit outcrops in the study area. In particular, the landslides primarily affected surficial soil cover on gentle slopes made up of clays and marly clays (60%) and secondarily flysch (27%) and alluvial or debris deposits (13%).

The surveyed effects were inventoried according to the criteria established for the Italian Catalogue of Earthquake-Induced Ground Failure database^53^ (CEDIT), which did not document evidence of past earthquake-induced ground effects in the study area. Only a few landslides and ground cracks are referred to the historical 1805 (Sant’Anna), 1627 (Gargano), 1894 (Lesina), and 1980 (Irpinia) earthquakes that took place in the surrounding area.

The analysis of frequency distributions of total landslides and of slope morphologies in the entire study area with respect to topographic metrics (i.e., elevation and slope) or lithology provides a summary of the susceptibility of an area to landslides^54^. The frequency distribution of the landslides available in the catalogue shows peaked unimodal right-skewed (positive kurtosis and skewness) distributions for both elevation and slope, with mode values equal to 200–250 m a.s.l. and 20°.

EqTLs show a unimodal distribution with peaks centred at lower values of 150–200 m a.s.l. and a gradient of 15°. This finding marks the importance of seismic input in controlling landslide occurrence (i.e., the observed scenario) since landslides occur even at slighter slopes and lower elevations (consistent with those that characterise the epicentral area).

Based on the first analysis reported in Martino et al.^26^, the surveyed EqTLs also show a slight NE-SW directivity in their spatial distribution in accordance with the main morphostructural alignments, such as the fluvial and drainage networks, which represent features of the morphostructural setting.

This directivity seems to be more pronounced for first-time landslides than for reactivations^26^. However, to date, the distribution of EqTLs does not provide evidence supporting the link between the directivity and the strike-slip fault mechanism. Moreover, regarding the distribution with respect to the epicentral distance, a typical decreasing frequency of EqTLs is observed with increasing distance, with an almost symmetrical distribution, especially parallel to the strike of the reliable fault plane (Fig. 1).

Maximum distances of 7 km for disrupted landslides (i.e., mainly represented by rock falls) and of 18 km for coherent landslides (i.e., earth landslides) were surveyed. Based on these epicentral distances, given the magnitude of the earthquake, all the disrupted landslides occurred below the maximum expected distance provided by Keefer^6^ on world-based statistics and by Martino et al.^55^ for the Italian territory. In contrast, 43 out of 75 coherent landslides took place beyond the maximum expected distance, located approximately 3.5 km from the epicentre. As inferred by Martino et al.^26^, the occurrence of coherent landslides beyond the established thresholds can be attributed to the coupled and quasi-simultaneous action of preparatory factors (i.e., rainfall) and triggering factors (i.e., the earthquake). The unexpected distance of occurrence with respect to the Keefer affected area, which is based on the registered magnitude of the event, allows delineation of the perturbed area, where possible evidence of increased postseismic activity can be assumed.

**Figure 2:**
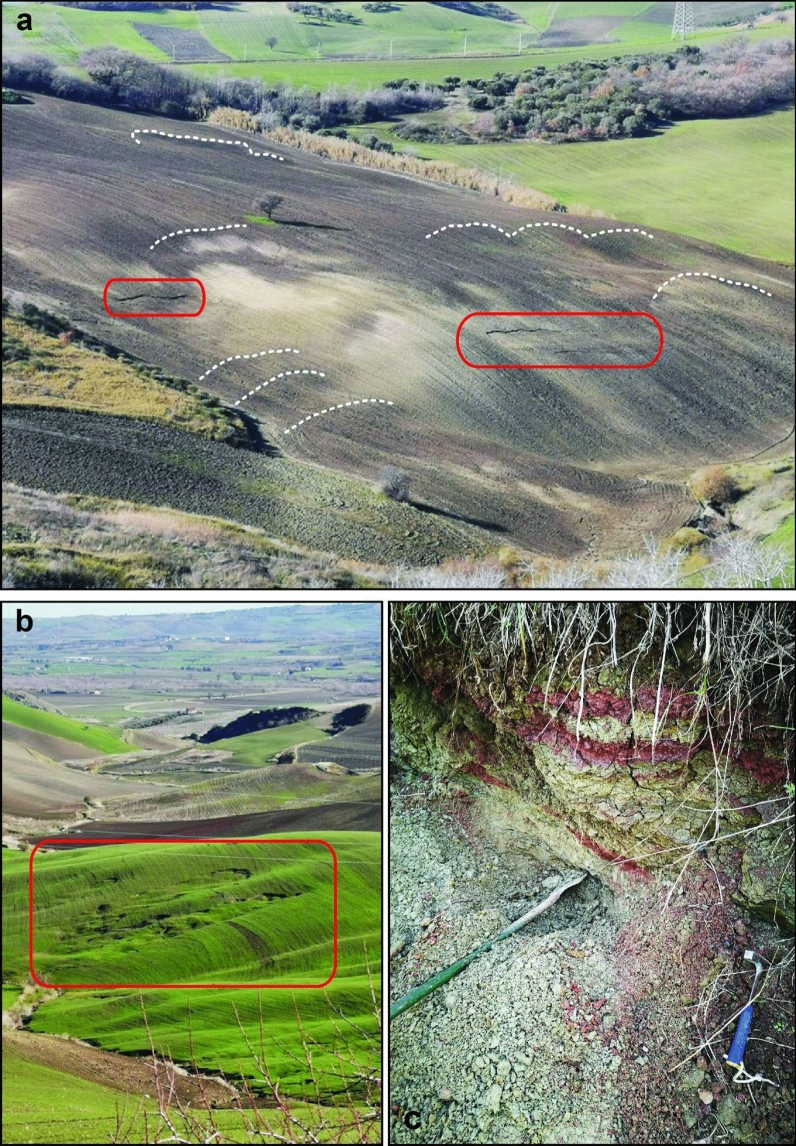
(**a**,**b**) Pictures of exemplary postseismic landslides (red boxes) recognised in the field, showing evidence of past scarps of landslides. (**c**) Example of scaly clays that crop out widely in the study area. Pictures were taken by the Authors.

Despite the low magnitude of the seismic event, the combination of preparatory and trigger factors (i.e., rainfall and seismic shaking) resulted in an increased impact on the territory, as testified by the surveyed scenario^26^. These destabilising factors acted on mainly clayey (both blue and scaly clays belonging to the former pelagic Molisan Basin^56^) and flysch lithologies, characterised by very low friction angles^42,57^ (lower than 22°) resulting in gentle slopes, affected a priori by abundant landslides. The latter show reactivations every year involving shallow soils and eluvium-colluvium covers (Fig. 2). The illustrated morphologies devoid of vegetational or anthropic disturbance elements and featuring abundant landslides has already been revealed to be proper for investigating landslide triggering using DInSAR analysis^58^. For these reasons, evidence of reactivations or first occurrence of landslides was monitored over a medium term consisting of two years before the seismic event and the first year after the mainshock to assess how even relatively weak seismic events can affect the spatial and temporal distribution of landslides in predisposed areas.

In the following section, evidence or proxies of increased activity have been recorded and are discussed within the framework of the seismotectonic, geo-lithological and morphostructural setting of the area.

## Results

To evaluate landslide activity in the study area after the Mw 5.1 Montecilfone earthquake (Italy), a multitemporal analysis of satellite SAR images over three years across the seismic event was conducted. Evidence of variations in the landslide rate in the postseismic year was investigated and compared with that for “unperturbed” conditions during the two years before the earthquake.

In more detail, pivoting the analysis on the date of Montecilfone earthquake (i.e., August 2018), two years before the earthquake (September 2017–August 2018—i.e., the 1st preseismic year—and September 2016–August 2017—i.e., the 2nd preseismic year) and one year after the earthquake (September 2018–August 2019) were scanned in detail by means of DInSAR. In total, 350 interferograms covering a time span between October 2nd, 2016, and August 1st, 2019, were exploited to detect both the reactivation of pre-existing landslides (already mapped in the IFFI catalogue) and first-time inventoried landslides in the two years before and in the year after the Montecilfone mainshock. Interferometric fringes related to reactivation of pre-existing landslides or first-time landslides (sensu Hutchinson^52^) were accounted for based on the comparison with the available landslide catalogues.

**Figure 3:**
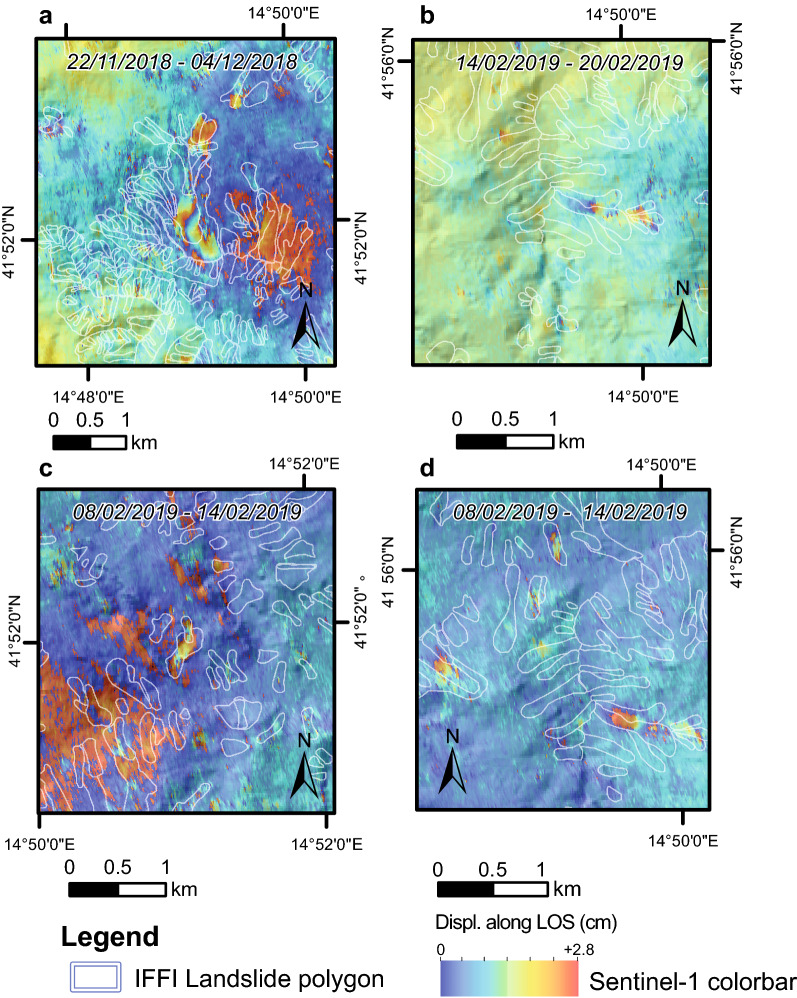
Examples of landslide reactivation from Sentinel-1 interferograms; each colour fringe from blue to red corresponds to 2.8 cm of displacement. White lines indicate the landslide perimeter from available catalogues. Images Satellite images available from interferometric Copernicus Open Access Hub (https://scihub.copernicus.eu/dhus/#/home) and processed by SARPROZ © tool (academic licensee Sapienza).

**Figure 4:**
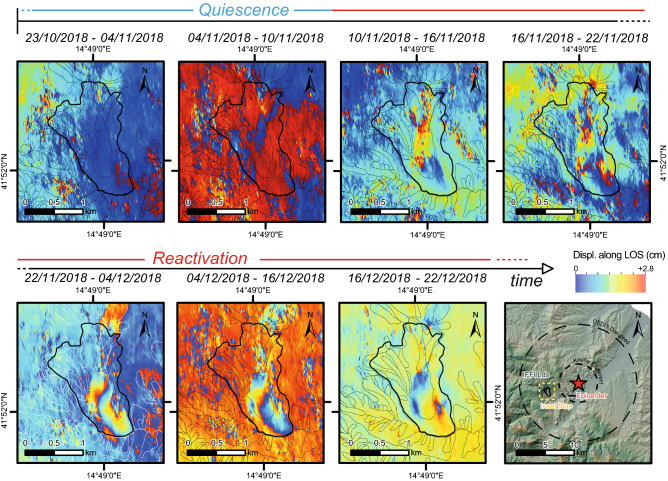
Time series of interferograms showing the reactivation of a landslide the study area (contour of the landslide in black, and location in the last panel) from October 23rd, 2018 to December 22nd, 2018. The last five interferograms refer to a single reactivation. The occurrence of fringes can be observed with respect to the quiescence stage, represented by the first two interferograms. Satellite images available from interferometric Copernicus Open Access Hub (https://scihub.copernicus.eu/dhus/#/home) and processed by SARPROZ © tool (academic licensee Sapienza).

Some examples of landslide reactivations are represented in Fig. 3, where four interferogram subsets highlight the correspondence of interferometric fringes with already mapped landslides and properly describe how the ground deformed in the time span covered by the interferograms. Moreover, in Fig. 4, an example of a time series of displacement for one of the largest landslides of the study area is shown. The time series is represented by a sequence of interferograms encompassing an initial period of quiescence that evolves in a reactivation phase. In particular, in the interferograms from 10/11/2018 to 16/11/2018, the first small interferometric fringes appear inside the landslide body, showing the start of the reactivation phase. In subsequent interferograms the fringes become clearer, with a continuous and well-defined shape, involving a larger area (in the interferograms from 22/11/2018 to 04/12/2018 and from 04/12/2018 to 16/12/2018 two complete fringes are shown). From November 22th to December 16th 2018, indeed, the ground deformation phenomena achieve the maximum velocity rate for this reactivation event, and it begins to decrease in velocity in the last interferogram represented in Fig. 4.

A significant increase in the number of landslide reactivations was detected in the epicentral area, especially in autumn and winter of the postseismic year, corresponding to 308.6 mm of rainfall. This value is of the same order of magnitude as those recorded in the same periods of the years before the earthquake, where 340.8 and 300.0 mm of rain fell in 2017–2018 and 2016–2017, respectively. Therefore, seasonal rainfalls are completely comparable over the three years of investigation, suggesting that they did not act as a preponderant and exceptional destabilising factor for landslides increasing in postseismic seasons. Table 1 summarises the polygons corresponding to reactivated landslides retrieved by interferometric analysis before and after the Mw 5.1 Montecilfone earthquake. In particular, considering the number of landslide reactivations, a positive percentage variation equal to + 118% was determined for the postseismic year with respect to the 1st preseismic (2017–2018) year, while an increase equal to + 54% was determined for the postseismic year with respect to the 2nd preseismic year (2016–2017). These high values of increase reveal that there was a sharp increase in the number of landslides during the year following the earthquake. These results are also confirmed by considering the absolute number of landslide polygons where displacement was detected, regardless of the number of reactivations (Table 1). In this latter case, increases in the numbers of reactivated landslide polygons during the postseismic year equal to + 87.5% and + 45% were found compared with the 1st and 2nd preseismic years, respectively.

**Table 1:** Table reporting the number of total landslide reactivations (bold) and the number of reactivated polygons (italics in brackets) for each considered year and for both IFFI landslides and first-time landslides. The number of surveyed EqTLs immediately after August 16th, 2018, is also reported.

Years	Before Montecilfone earthquake time		Coseismic (field survey)	After Montecilfone earthquake time
	2016–2017	2017–2018	16 August 2018	2018–2019
No. of landslide reactivations (pre-existing landslides reported by IFFI catalogue)	**139** (*101*)	**103** (*83*)	*33*	**174** (*132*)
No. of first-time landslides (by interferometric analysis)	**28** (*23*)	**15** (*13*)	*51*	**83** (*48*)
Total	**167** (*124*)	**118** (*96*)	*84*	**257** (*180*)

Tables 2 and 3 report the number of landslide reactivations for both IFFI polygons and first-time landslides over the seasons. The relevant increases in landslide reactivations in the postseismic year occur mainly in the autumn and winter seasons. More specifically, in autumn 2018, we noticed a positive percentage variation up to + 2566% with respect to the preseismic autumns, while in the postseismic winter, we registered a positive percentage variation up to + 60% with respect to the preseismic winters. The most significant increase in landslide number, compared to the same period of previous years, occurred in the three months (i.e., Autumn 2018) immediately following the Montecilfone earthquake, suggesting that the seismic event contributed to reducing the resistance of the soils and rocks outcrops. Furthermore, the increase in the number of postseismic landslides took place net of seasonal rainfall, which in autumn 2018 had comparable or lower values than the same season in the previous two years: 308.6 mm of rain in autumn 2018 versus 340.8 mm of rain in autumn 2017 and 300.0 mm of rain in autumn 2016.

**Table 2:** Table reporting the number of total landslide reactivations for both IFFI landslides and first-time landslides, divided according to the seasons and for each considered year.

Years	No. of landslides reactivations over the seasons				
	Autumn	Winter	Spring	Summer	Total
2016–2017	4	121	42	0	167
2017–2018	3	91	24	0	118
2018–2019	80	146	31	0	257
					542

**Table 3:** Table reporting the percentage variation in the number of total landslide reactivations recorded in each season compared to the same season of the year before.

Years	Percentage variation in landslides reactivations between the same seasons of subsequent years					
	Autumn		Winter		Spring	
2016–2017 (2nd preseismic)	4	**1900%**	121	**21%**	42	**− 26%**
2018–2019 (postseismic)	80		146		31	
2017–2018 (1st preseismic)	3	**2566%**	91	**60%**	24	**29%**
2018–2019 (postseismic)	80		146		31	

Furthermore, some additional landslides were detected by DInSAR in only the postseismic year, which were not present in the official databases and could therefore be assumed to be first-time landslides.

An increase in landslide activity in the postseismic period also resulted in landslides with continuous activity (i.e., flow-type landslides); given this result, continuous activity was assumed if there were no interruptions lasting through at least three consecutive interferograms. In fact, as revealed by the DInSAR analysis, these landslides showed a significant increase in the number of days of movement in the postseismic year with respect to the number of days of movement detected in the two analysed preseismic years. This increase was on average equal to + 57 days (as a positive change between the number of days of movement detected in the postseismic year and the number of days of movement detected in one of the preseismic years) of continuous activity compared to the 2017–2018 preseismic year and equal to + 17 days of movement compared to the 2016–2017 year, reaching increases of + 120, + 140, and + 150 as the changes (Δ) in the number of days of movement for some landslides. The most common mechanism in continuous-type landslides is earthflows (17 landslides), which mainly involves the scaly clays and Faeto flysch formations, and 6 landslides have a complex type of movement. These categories of landslides often originate in clays with an earth slide mechanism where a flow involves landslide deposits at the slope foot. This is the case for the landslides that show the greatest increase (Δ) in the number of days of movement in the postseismic stage compared to the preseismic stages (+ 150 days). Sliding and fast-flow landslide mechanisms are less common among continuous-type landslides (i.e., 2 and 1 inventoried landslides, respectively).

To better assess the destabilising role of the earthquake in increasing landslide activity in the postseismic year in the epicentral area, a landslide density spatial analysis (Fig. 4) was carried out over the three years in a regular square 1.5-km fishnet to establish the concentrations of landslide reactivation for both IFFI and first-time landslides over the three years considered. The density of the landslide reactivations per cell in the postseismic year was greater than those recorded for the two preseismic years, covering an area around the epicentre that is consistent with the distance where EqTLs occurred^26^ as well as with the PGA scenario. In particular, the landslide reactivation density derived over all fishnet cells reported the maximum (red and orange cells) and medium (yellow cells) concentrations in accordance with the shape of the shakemap obtained for the mainshock of August 2018 and with the expected distances of EqTL occurrence^6,59^. As reported above, an anomalous increase in the landslide reactivation number was observed during the autumn and winter seasons in the postseismic year. Since the pluviometric regime of autumn and winter after the 2018 earthquake is comparable to those recorded in the same seasons of the years preceding the 2018 earthquake, as described in the following Methods section, we note that the increase in the number of reactivations of landslides is independent on the rainfall but closely linked to the destabilising action that the earthquake caused in the soils outcrops, which reduced the resistance in the medium-long term and would have acted as a preparatory factor for the rainfall-induced reactivations experienced in the postseismic interval.

Based on the focal mechanism solution of the 2018 Molise earthquake returned by the National Geophysical and Volcanology Institute (INGV) and given the tectonic settings of the area, the N80°W-oriented dextral transcurrent solution may be assumed to be the seismogenic element source of the Mw 5.1 earthquake (Doglioni C., 2021, personal communications). The relationship between the orientation of fault slip that generated the earthquake and the landslide occurrence discussed for EqTLs by other Authors^15,54^ was also evaluated in the postseismic stage, with the aim of highlighting potentially irreversible modifications in the stability of slopes and landslide activity ascribable to fault mechanisms or directivity of ground motion.

To observe the relative distribution of the landslides as a function of the distance from the tectonic elements parallel and perpendicular to the fault strike, two mutually perpendicular transects were reconstructed. These transects were computed for both the preseismic years and the postseismic year. The transects were constructed by projecting all the landslides surveyed across the considered tectonic lineament within a buffer 1.5 km wide on both sides of the two traces, covering the whole epicentral area for a distance greater than that of the Keefer curves for disrupted landslides^6^. More specifically, to evaluate the landslide distribution over time, the total number of reactivations of each landslide was considered.

**Figure 5:**
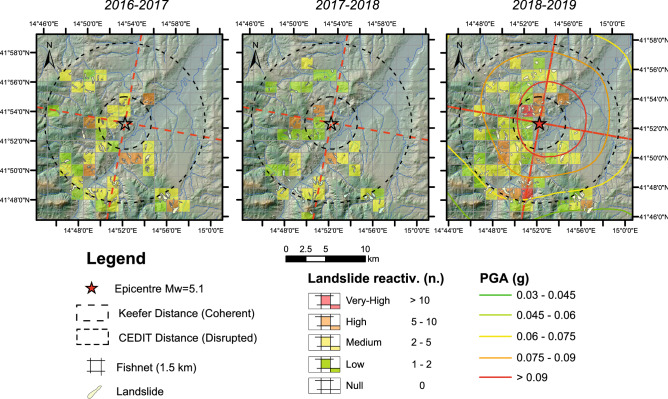
Spatial distribution of landslide reactivation density derived over a regular square 1.5-km fishnet during the three years before and after the August 16th, 2018, Montecilfone earthquake*.* Map is created by ArcGis Desktop 10.8 (Licensee Customer Number: 274576—Sapienza University) using a hillshade model from the open source Shuttle Radar Topography Mission (SRTM) 1 Arc-Second Global DEM (https://earthexplorer.usgs.gov/).

**Figure 6:**
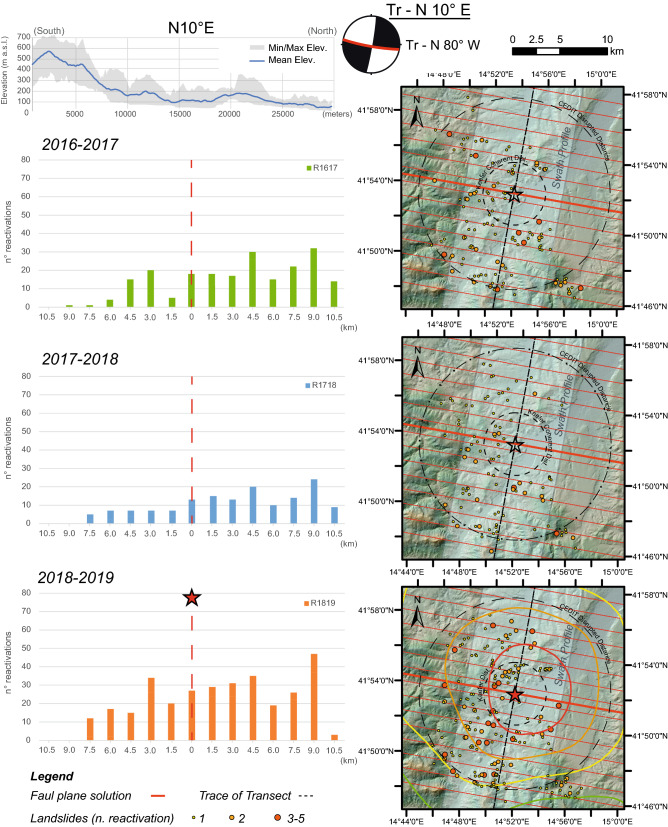
Distribution of landslide reactivations over 2016/2017 (**a**), 2017/2018 (**b**), and 2018/2019 (**c**) in directions perpendicular to the fault strike. Histograms of reactivations over a 1.5 km-wide buffer along transects are also reported. Stars indicate the epicentre location. Map is created by ArcGis Desktop 10.8 (Licensee Customer Number: 274576—Sapienza University) using a hillshade model from the open source Shuttle Radar Topography Mission (SRTM) 1 Arc-Second Global DEM (https://earthexplorer.usgs.gov/).

Figure 5 shows the three distributions obtained along the N10°E section, oriented perpendicular to the fault plane, projecting the position of landslides along the WNW-ESE direction. An almost symmetrical distribution in the number of reactivations across the tectonic element is observed for the two preseismic years. The number of landslide reactivations in the 2 years preceding the earthquake, regardless of the position along the transect, is below that observed in the postseismic year. In the postseismic year, a homogeneous increase in the number of reactivations is observed for all band increments across the fault, reflecting the same trend registered in previous years. No marked asymmetry is registered across the fault plane (Fig. 6).

**Figure 7:**
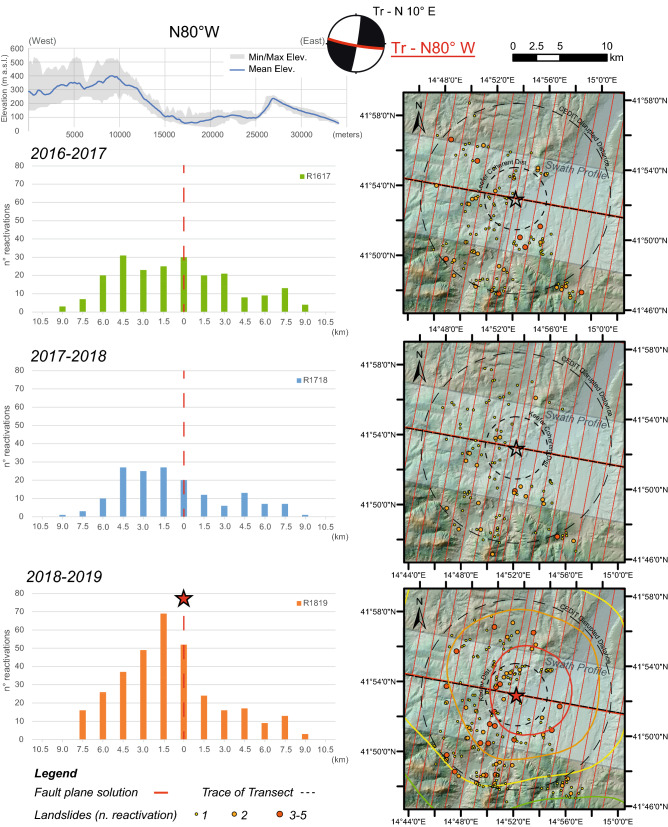
Distribution of landslide reactivations over 2016/2017 (**a**), 2017/2018 (**b**), and 2018/2019 (**c**) in directions parallel to the fault strike. Histograms of reactivations over a 1.5 km-wide buffer along transects are also reported. Stars indicate the epicentre location. Map is created by ArcGis Desktop 10.8 (Licensee Customer Number: 274576—Sapienza University) using a hillshade model from the open source Shuttle Radar Topography Mission (SRTM) 1 Arc-Second Global DEM (https://earthexplorer.usgs.gov/).

Conversely, the distribution of landslide reactivations in the postseismic year projected onto a transect parallel to the fault alignment reveals an asymmetrical distribution. In fact, in the W sector, where compression occurred, the number of landslide reactivations is up to three times greater than that of landslides reactivated in the E sector. The linear distribution of the number of reactivations also indicates a sharp asymmetric and decreasing trend moving away from the epicentre (Fig. 7). An even greater increase in landslide activity was recorded after the seismic event with respect to the total effects activated over the years 2016–2017 and 2017–2018 (Fig. 7).

No relevant changes in topographic elevations and gradients are retrieved along the analysed transects. The distribution in the number of reactivations is observed over an alignment characterised by a homogeneous gentle topography with variable heights ranging between 200 and 400 m a.s.l. and low gradients ranging between 10° and 20°. As revealed by the swath profile, a nonnegligible change in morphological metrics (elevation and slope roughness) can be observed on the right bank of the Biferno River towards the NE sector of the area, where a flat valley filled by alluvial deposits is present; thus, this sector should not be considered for a more consistent spatial analysis.

## Discussion

The results of the DInSAR analysis over a three-year time interval encompassing the seismic event of 2018 highlight the landslide style and distribution of the reactivations before and after the earthquake and during the almost simultaneous pluviometric event, considered a common rainfall pattern^26^.

In general, autumn and winter, i.e., the wettest seasons, are characterised by the greatest number of reactivations of landslides or by their first occurrence since rainfall acts as a trigger following the saturation of the shallow parts of the lithotypes that crop out. The DInSAR analysis and the available databases of landslides allow us to estimate the number of reactivations of already existing landslides and the number of first-occurrence landslides (i.e., not included in the databases) in the study area. The most represented types of movement are slow earth landslides, such as flows and planar or rotational slides, mainly involving scaly or marly clays and the Faeto flysch.

Within this susceptible geological and morphological setting, the low-magnitude earthquake and the relatively weak motion (expressed by the PGA map in Fig. 1) would have destabilised and prepared the soil cover and the existing landslide deposits to evolve at a higher rate under similar rainfall intensity^60^. The spatial distribution of the landslide reactivations in the postseismic year marks the epicentral area and reflects the maximum PGA (g) values (0.06–0.075 g up to 0.12 g). This confirms that the seismic shaking experienced by soil could affect the stability of the slope and the equilibrium of pre-existing landslides.

During the two years before the earthquake, when cumulative rainfall amounts in the wetter seasons were similar, the number of reactivations or first-time landslides are quite comparable in terms of absolute numbers. In contrast, a marked difference in the number of reactivations is noted in the postseismic year, with an increase of 118% considering both the reactivated IFFI polygons and the first-time landslides. In this context, the increased landslide activity following the earthquake was not consequent to a high-magnitude event, but the temporarily accelerated slope erosion posed by landslides is attributed to the combination of lithologically predisposed slopes and the simultaneous effects of rainfall and low Mw 5.1 seismic shaking. In addition, most of the landslides were coherent in type, and most mechanisms were characterised by slides and slow earth flows, while generally after an earthquake, the most common landslides were debris flows^61–63^.

In the case of high-magnitude events, this increase in activity is expected to be recovered in the following years^1^. In this study, a trend towards transient recovery was observed as early as the postseismic winter. The percentage seasonal increase shows that this was concentrated in the autumn following the mainshock, where the positive variations reached + 2566% and + 1900% with respect to the same season in the 1^st^ preseismic and 2^nd^ preseismic years, respectively. A decreasing trend of landslide activity and a recovery to preseismic conditions were registered from three to six months after the mainshock, as shown by lower increments (+ 60% and + 20% with respect to 2018–2017 and 2017–2016). The observed effect appears negligible in the postseismic spring, i.e., more than six months after the earthquake, since the number of reactivations is comparable to that observed in the two years before the earthquake, suggesting how even small earthquakes can lead to rapid changes in slope erosion rates in the clayey hillslopes considered here.

The potential contribution of anomalous rainfall as a trigger in landslide activity in the months immediately following the earthquake can be excluded because the area had a similar pluviometric regime over the three years considered. More specifically, in autumn and winter of the postseismic year, i.e., in the rainy season, the total rainfall amounts are lower than those of the 2 years preceding the earthquake, with 463 mm and 542 mm accumulating in six months. Therefore, the rains after the 2018 earthquake cannot be considered the main cause of the exceptional increase in landslides in the postseismic period. Conversely, the earthquake is retained as an aggravation factor that contributed to the rainfall-induced scenario observed in the first postseismic year, playing a leading preparatory role in the slope disequilibrium.

To better frame the landslide style of activity before and after the Montecilfone seismic event, a systematic analysis of the state of activity of the reactivated landslides from 09/2016 and 09/2019 was carried out by DInSAR analysis. The total days of activity and the inactivity period were computed as the difference between the first and the last subsequent interferograms where a landslide shows evidence of movement (i.e., clear interferometric fringes) and the time between two different reactivations (e.g., monthly, seasonal, annual, or pluriannual). To be consistent, all the time differences were computed based on the date of the master SAR images; thus, a systematic error in landslide timing equal to the revisit time (i.e., ± 6 days) was introduced.

The state of activity of the monitored landslides, expressed in terms of days in which movement is detected in consecutive interferograms and days of inactivity (i.e., timespan between two consecutive reactivations of the same landslide), is shown in Fig. 7. For both preseismic and postseismic times, four main types of landslide activity have been recognised and are classified into four groups:

- Group I: Seasonally recurrent landslides, with two or more reactivations in the same year (time of inactivity fewer than 150 days);
- Group II: Yearly reactivated landslides, single reactivation within a year, every 200–450 days;
- Group III: Continuous landslides with a long time of activity (movement detected for at least 45 days up to 200 days);
- Group IV: Landslides that were reactivated in only the postseismic year and were inactive for at least two years (time of inactivity greater than 500 days), i.e., which were reactivated at least before autumn 2016 and characterised by a single reactivation over one year.

**Figure 8:**
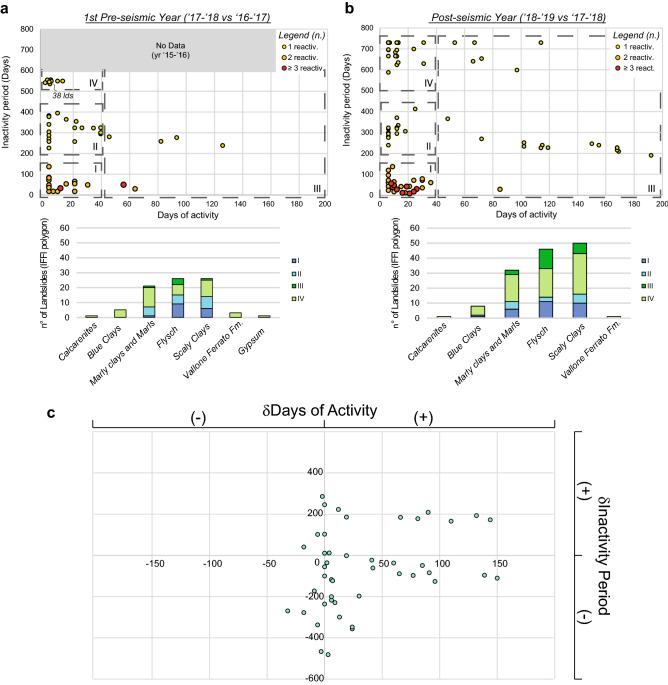
State of activity of surveyed landslides for both preseismic years (**a**) and the postseismic year (**b**) (top) with respect to the preceding years. Histograms relating landslides in the groups with the involved lithologies (bottom) are shown. A clear increase in the number of landslides and number of reactivations in postseismic time is visible. (**c**) Differential values computed between the postseismic year and preseismic years for the days of activity and inactivity period; for several landslides, an increase in their mobility in the postseismic period is visible (negative differential for the inactivity period and positive differential for the days of activity).

A comparison among the results obtained for the 1st preseismic year (Fig. 8a) regarding the 2nd preseismic (2016–2017) and the postseismic year (Fig. 8b) with respect to the 1^st^ preseismic year (2017–2018) reveals an increase in the number of reactivated landslides belonging to group IV, which showed no evidence of movement in the two preceding years. Moreover, an increase in the rate of activity of continuous landslides is expressed by an increase in the days of activity during the postseismic year with respect to the previous years. These reactivated landslides reveal an annual or multiannual recurrence and belong to group III. The greatest increase occurred in the postseismic year for the landslides belonging to group I, as they increased in the total number of landslide reactivations. Based on the comparison between the state of activity observed in the postseismic year and that detected in the preseismic years, we can state how the earthquake changed the style and activity of landslide processes, increasing them in absolute number (i.e., number of reactivated landslides), in the number of reactivations and in the duration of the activity.

An increase in the rate of landslide occurrence can be deduced by calculating the differentials of the two parameters that describe the state of activity (days of activity and inactivity period) of each landslide between the postseismic year and the preseismic years (Fig. 8c); this increase is evident for the postseismic year. Several landslides indeed show an increase in days of activity. At the same time, the inactivity period does not show systematic clusters, perhaps due to the role of seasonal rainfall as a trigger.

Regarding the deposits involved in the postseismic landslide activations (histograms in Fig. 8), the derived distribution indicates an increase in the number of reactivated landslides, especially for flysch and scaly clays. Together with the marly clays and marls, these lithologies represented the lithologies most involved in landslides, even in the preseismic period, marking the susceptibility of the study area to slope failures after relatively low-magnitude earthquakes.

Compared with the rates of landslide movement obtained by DInSAR analysis for the two preseismic years considered (in the autumn and winter seasons), the landslide movement rates increased in the postseismic year, especially in the three months following the mainshock. Given the registered trend of reactivations, the enhanced rates of landslides recorded after the earthquake might have been driven not only by rainfall or aftershocks but also by the lowered strength of slope soil materials (i.e., loosening of regolith or soil covers) due to coseismic mainshock shaking, which occurred despite the low magnitude of the 2018 Montecilfone earthquake (i.e., Mw 5.1). The recovery of stability within a few months following the earthquake could be related to the low magnitude of the mainshock; literature data reveal a possible inference of the earthquake magnitude on the occurrence rate over a decade, as the time span for stability recovery varies from 1 year up to 10 years for earthquakes with Mw greater than 6.6^1,7,64^.

Earthquake-induced failures can involve eluvium, regolith and bedrock with different mechanisms^1^. For eluvium and regolith, mechanical damage and ground crack opening could affect the postseismic landslide rate, while for bedrock, joints or bedding planes can form or widen during shaking but influence medium- to long-term slope stability after the earthquake. The postseismic landslides presented here mainly involve outcrops of the shallow soil covers (i.e., regolith example presented in literature) that would have experienced gravitational disequilibrium, thus justifying, together with the low magnitude of the seismic event, an increased landslide rate in only the first months after the mainshock.

The increasing number of postseismic landslides also reveals an asymmetrical distribution with respect to possible solutions of the right-lateral strike focal mechanism related to the mainshock. In this regard, the dextral strike-slip fault solution, oriented N80°W, would have controlled the distribution of spatial disequilibrium, promoting landslide reactivations in the compressive sector. The fault rupture mechanism is one of the factors that controls the spatial distribution of the EqTLs along the ruptured tectonic element because earthquakes with unilateral fault ruptures present shaking that can be asymmetric along the fault^1,65–68^. In general, different types of seismogenic faults can induce several EqTL patterns^15,69^. Coseismic landslides triggered by strike-slip faults show a more symmetric distribution across the fault and are distributed in a narrow area^70,71^, unlike reverse or normal fault mechanisms, whose spatial density distribution is more concentrated on the hanging wall^65,66,72–74^. This last feature was also observed for the Italian territory following the 1980 Mw 6.9 Irpinia earthquake, in which an asymmetric distribution of EqTLs characterised by a higher concentration of slope failure on the hanging wall of the seismogenic normal fault was surveyed^55^. In the case study presented here, both EqTLs (i.e., coseismic) and postseismic landslides show asymmetric distributions, considering the frequency distribution along a transect parallel to the fault strike, with a number of landslide reactivations greater in the W sector and with a maximum near the fault rupture (Fig. 7). In contrast, the distribution of coseismic effects and landslide reactivations over years in directions perpendicular to the fault strike shows an almost symmetric distribution of both EqTLs and postseismic landslides.

**Figure 9:**
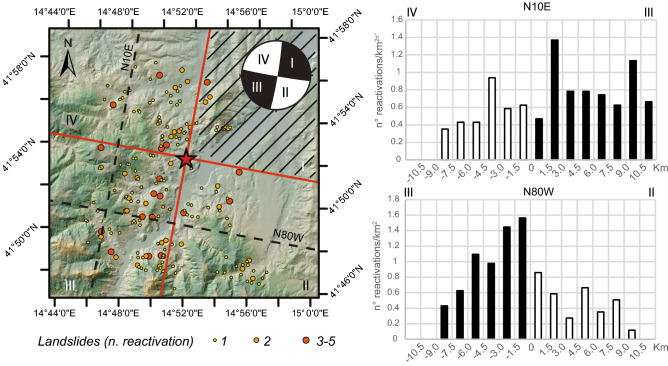
Distribution of landslide reactivations in directions perpendicular (N10°E) and parallel (N80°W) to the fault strike for both extensional and compressional zones (landslide frequency normalised for extension vs. distance to the fault). The NE sector, identified with the oblique black lines, was not analysed in the statistics because of the topographic peculiarities. Map is created by ArcGis Desktop 10.8 (Licensee Customer Number: 274576—Sapienza University).

An asymmetric distribution (expressed in terms of both spatial density and number of reactivations) of postseismic landslides was also observed in the compressional and tensional zones of the fault rupture. Considering the transect perpendicular to the fault (transect oriented N10°E), an almost symmetric distribution of reactivated landslides can be identified, with a slight prevalence in the compressional zone (Fig. 9). In the direction parallel to the fault (oriented N80°W), in contrast, a marked asymmetry is evident, again in favour of the compressive sector, with the greatest number of landslide reactivations in the postseismic year close to the tectonic element and decreasing with the distance from it (Fig. 9). According to what it was argued in the previous paragraph, Fig. 9 marks the NE sector of the study area that was not included in the distribution analysis of the EqTLs, due to the peculiar topography which corresponds to the Biferno River plain, i.e. not comparable with the hill slopes where the landslides occurred.  In Italy, a similar asymmetric distribution was observed in coseismic stages after the Mw 6.9 Irpinia earthquake of 1980, with a majority of EqTLs surveyed in the hanging wall block^55^. These outcomes mark and highlight the importance of the fault mechanism for driving not only the distribution of earthquake-triggered landslides (i.e., the pattern of slope failures observed immediately after the event or up to two days after) but also the style and rates of landslide activity in the postseismic years, depending on the earthquake magnitude and a priori landslide susceptibility of the study area. Although the role of the earthquake fault mechanism in the distribution of postseismic landslide activity is far from fully understood, seismic shaking would have contributed to the deferred instability and acted as a preparatory factor on the involved slopes.

The methodology applied here highlights the suitability of satellite DInSAR for detecting impulsive phenomena such as landslide reactivations and nonlinear movements such as the acceleration of a landslide, representing a more suitable approach than advanced multitemporal techniques (A-DInSAR). Remote surveying has proven to be a very effective tool for both evaluating landslide scenarios after a mainshock and discriminating causative or predisposing factors that control the landslide distributions in a long-term window, i.e., several years after the seismic event.

## Conclusions

Quantifying variations in the state of landslide activity after earthquake occurs is a challenging topic that requires the coupling of field-collected and remotely sensed data. Based on the time period of landslide activity as well as on the duration of inactivity intervals, the nonnegligible effects of earthquake coseismic shaking and postseismic short-to-middle displacement time on landslide processes are highlighted. If force-driven effects due to dynamic actions can be hypothesised and conventionally quantified through pseudostatic or pseudodynamic approaches, the same is not possible for creep-driven phenomena such as earthflows. In these cases, the inference of soil moisture and seasonal saturation of the involved debris covers requires a high number of episodes to perform a reliable statistical analysis of earthquake-induced effects. In this regard, the selected case study offers a rich database to be analysed in terms of both the spatial distribution and time recurrence of landslide events.

The remote survey presented here highlights the intensification of landslide activity after a low-magnitude earthquake, which struck slopes featuring very high susceptibility. This transient increase involved the first year after the earthquake. The outcomes obtained here indicate the relevance of combining preparatory and triggering factors in landslide scenarios, which can be relevant in both coseismic and postseismic stages, as retrieved by the analysis of landslide reactivation in the postseismic period.

Based on such an analysis, the inference of tectonic and faulting mechanism evident in the coseismic interval, usually surveyed by EqTL distribution, is also evaluated for the postseismic stage, where an asymmetric distribution and a relevant concentration of landslides are registered in compressive sectors parallel to a strike-slip faulting mechanism.

This study demonstrates that low-magnitude earthquakes can also play a morphogenetic role in the framework of landscape evolution over short to moderate time periods and proposes a quantitative analysis to output landslide activity variations at a basin scale after an earthquake. Moreover, the approach proposed here, which integrates direct field observations with remotely sensed observations, is promising in terms of reliability and coverage of spatial information.

## Materials and methods

### Multi-temporal DInSAR analysis

In total, 332 C-band Sentinel-1 SAR images were acquired for a time interval between October 2nd, 2016, and August 1st, 2019, consisting of 167 images in ascending orbital geometry and 165 images in descending orbital geometry, with a revisit time of 6 or 18 days and spatial resolution of 5 × 20 m, in interferometric wide-swath mode (Table 4). More than 350 interferograms were processed over an area of approximately 1200 km^2^ based on the differential synthetic aperture radar interferometry (D-InSAR) technique using SARPROZ software.

**Table 4:** Details of the satellite interferometric data collected.

Sentinel-1	Ascending	Descending
No. of scenes	167	165
Start date	2-Oct-2016	1-Aug-2019
End date	2-Oct-2016	1-Aug-2019
Look angle (°)	39.05	38.96

DInSAR is a radar-based technique that exploits the information contained in the phase of at least two SAR images acquired over the same area at different times to obtain interferometric differential maps (namely, “interferograms”^75^). To achieve reliable displacement results, the topographic component was detected and subtracted from differential interferograms. The 30 m-resolution digital elevation model (DEM) from the Shuttle Radar Topography Mission (SRTM) was used to remove the topographic component from the generated interferograms. The possible atmospheric disturbance on the single interferograms was not filtered, as this type of signal (a long spatial wavelength disturbance that affects large areas of the interferogram) does not affect the pattern of localised landslide fringes.

The areas affected by superficial ground deformation were represented by so-called interferometric fringes, i.e., bands of colour from blue to red, which corresponded to a ground displacement of 2.8 cm each (for C-band sensors).

The availability of both ascending and descending images allowed us to exploit redundant and independent datasets. The first step of the applied procedure was to fully screen each interferogram to detect spatial phase variations located on the slopes of the whole study area. Once a spatial phase variation was detected, i.e., interferometric fringes were observed, possible evidence of slope deformation could be preliminarily mapped and compared with subsequent interferograms and landslide inventories in the literature.

To drive the investigation of first-time landslides or reactivations of pre-existing landslides, the Italian Landslide catalogue was adopted as a first directory database. In object-oriented research, a single polygon mapped in the official catalogue was considered for counting the number of reactivations. More than 500 reactivations were registered, with locations or volumes of failures that partially or totally involved the pre-existing landslide perimeter available in the latest release of the IFFI catalogue (https://idrogeo.isprambiente.it/app/).

For these polygons, lithological information was extracted from the geological map at a 1:50,000 scale. The mechanism of landslide reactivation cannot be identified by interferogram analysis alone, and the possible differences with respect to the first-time landslide mechanisms remain uncertain. Nevertheless, field surveys can help highlight how reactivations involve soil covers and coherent landslide deposits in shallow or roto-translational mechanisms.

The deformation fringes for some landslides are present on most subsequent interferograms, suggesting continuous deformation over time, i.e., a flow-type landslide mechanism. In contrast, other landslides show discontinuous displacement patterns over time that represent different successive reactivation events. To count the number of landslide reactivations, the following general assumption was used: a new reactivation event was considered when a landslide showed deformation fringes after at least 3 successive interferograms (that corresponded to approximately 18 days) without any fringes. This systematic approach allowed us to count intermittent reactivations for some of the mapped landslides over both preseismic and postseismic years.

### GIS spatial analysis

To quantify the spatial distribution over time of the landslide reactivations, the density of EqTLs was derived over a regular square 1.5 km fishnet centred at the epicentral location; this method was previously adopted in Martino et al.^26^. The dimension of the fishnet density was selected based on the frequency distribution of the landslide polygon areas in the catalogue; a compromise between the fishnet resolution and the median size of the landslide, enveloping areas able to preserve the unique entity of a landslide polygon, was adopted.

The density of reactivations in the fishnet (Fig. 5) was calculated considering the number of reactivations over each considered year falling into the individual elements of the mesh. The calculated densities are non-dependent on the landslide size. The counting of the number of landslide reactivations was related to centroids of the landslide mass.

For morphometric and landslide distribution analysis, a 20 × 20 m DEM distributed by the Italian Ministry of Environment covering the entire Molise Region was adopted.

For the EqTLs (Fig. 1), variations in the number of landslide reactivation patterns along directions perpendicular to and parallel to the fault strike were evaluated in the postseismic time interval, along with N10°E- and N80°W-trending transects, respectively. The total landslide reactivations along the transect were calculated as the sum of the landslide reactivations per year within a buffer 1.5 km wide, which was equal to the fishnet mesh resolution, and were projected normally to the transect. To improve identification of possible changes in the morphological features of an area capable of discriminately predisposing the slope, altitude swath profiles^76^ were reconstructed along and normal to the fault direction. A swath strip width of 10 km was adopted to include 95% of the landslide reactivations that occurred in the area.

## Supplementary Information

Supplementary Information.
