# Supplementary material for: Increase in landslide activity after a low-magnitude earthquake as inferred from DInSAR interferometry
Source: Sci Rep. 2022 Feb 17;12:2686. doi: 10.1038/s41598-022-06508-w (PMC8854724; doi:10.1038/s41598-022-06508-w)
Supplement: Supplementary file 1 — Supplementary Information. [file 41598_2022_6508_MOESM1_ESM.docx]

Increase in landslide activity after a low-magnitude earthquake as inferred from DInSAR interferometry

S. Martino^1*^, M. Fiorucci^1^, G.M. Marmoni^1^, L. Casaburi^1^, B. Antonielli^1^, P. Mazzanti^1-2^

^1^Earth Sciences Department of “Sapienza” University of Rome and CERI - Research Centre for Geological Risk, P.le Aldo Moro n.5, I-00185 Rome, Italy. email corresponding author: salvatore.martino@uniroma1.it,

^2^ NHAZCA S.r.L., Spin-off Company of “Sapienza” University of Rome, Via Vittorio Bachelet n.12, I-00185 Rome, Italy

***Supplementary Materials***

*The analysis of pluviometric records in the epicentral area was conducted considering the daily rainfall recorded at Palata weather station, i.e., the closest rain-gauge to the epicentre of the seismic event. The cumulative monthly mm-rainfall have been achieved for the three-time intervals considered across the earthquake: from June 2018 to July 2017, from June 2017 to July 2016 (i.e., 1^st^ and 2^nd^ pre-seismic years) and from June 2018 to July 2019 (post-seismic year; Fig. S1).*


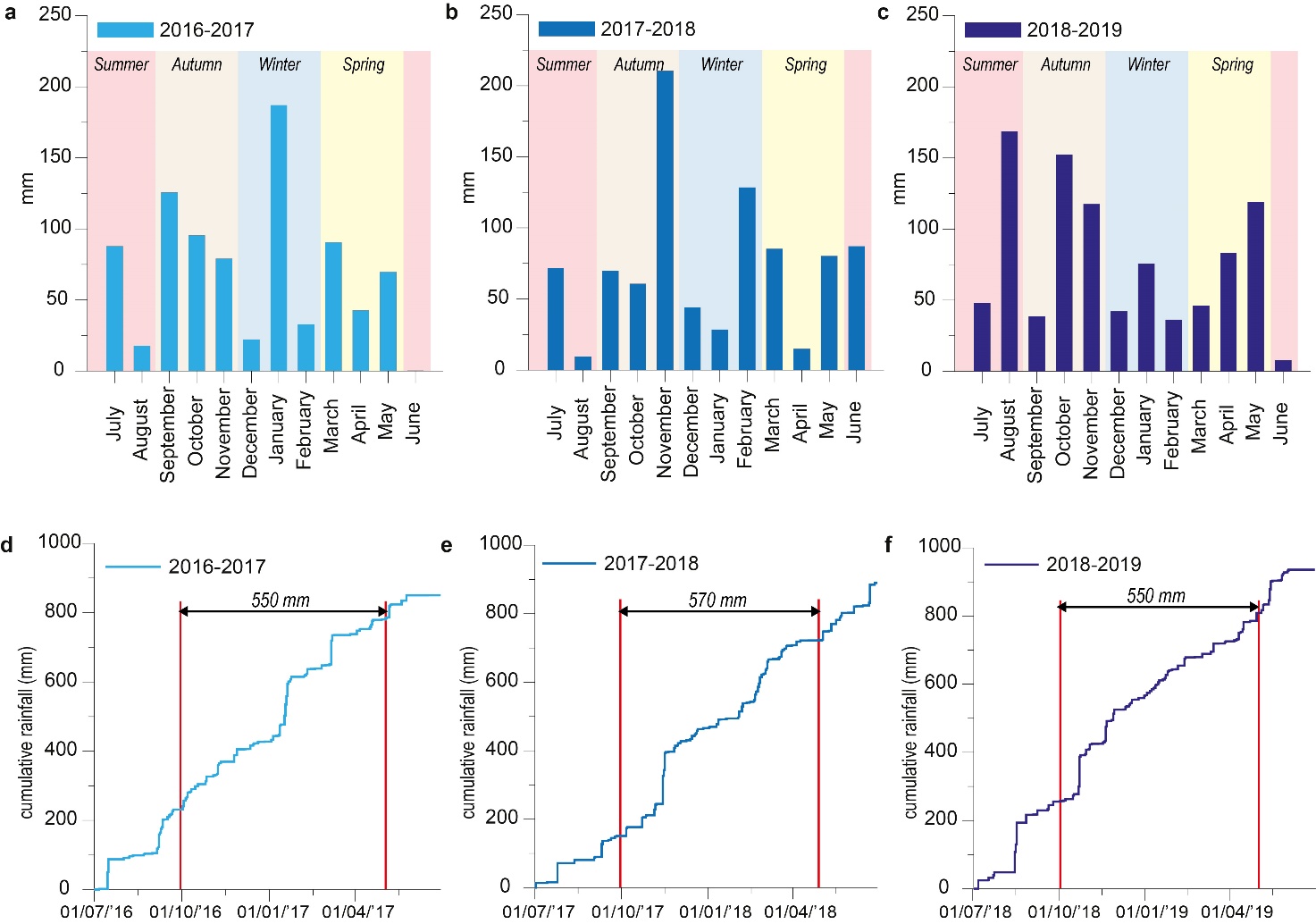


*Fig. S1: Histograms showing monthly rainfall over the three years of interest ( -a) 2^nd^ pre-seismic; -b) 1^st^ pre-seismic; -c) post-seismic) with the different seasons highlighted. Cumulated rainfall over the three years with the indication of mm fallen in landsliding seasons (from October to April -d) 2^nd^ pre-seismic; -e) 1^st^ pre-seismic; -f) post-seismic).*

*This analysis aims to verify the consistency of possible increases in the seasonal landslide occurrence, avoiding a main role of exceptional rainfalls in the increase of activity in landslides, verified by DInSAR analysis, in the months following the Montecilfone earthquake. To this purpose, the direct comparability of the monthly and seasonal rainfall values recorded in the Autumn and Winter after the earthquake with respect to pre-seismic years was verified, thus assuming that the pluviometric input played an equally important role in the three years considered.*

*To evaluate the exceptionality of the above-described rainfall fell in the three considered years, a statistical analysis of maximum daily and hourly rainfall intensity data was performed, exploiting the rainfall probability curves for the same study area. These curves have been reconstructed according to the Generalised Extreme Value (GEV) distribution considering rainfall data recorded by Ponte Liscione weather station, one of those closest to epicentral areas that guarantee historical time-series (since 1926). Given the short distance between the Ponte Liscione weather station and the Palata one (approximately 8 km from each other), we consider the GEV analysis also representative for Palata weather station, which does not present a historical series of the pluviometric data, and, therefore, of the whole study area.*

*The Return Periods (RP) of rainfall events responsible for the seasonal activation or reactivation of most pre-existing landslides (i.e., every Autumn) were calculated. Rainfall events before the landslides were taken into account and rainfalls were cumulated over a time span from 1 to 180 days.*

*The obtained results highlighted the low RP and thus the ordinariness of rainfall events responsible for the remotely surveyed scenario. The derived RPs, if referred to daily rainfall probability curves, show a wider period in the 2017-2018 (1^st^ pre-seismic) with respect to the 2018-2019 (post-seismic stage) (Table S1).*

|  | *Post-seismic* | | *1^st^ pre-seismic* | |
| --- | --- | --- | --- | --- |
| *Cumulated days* | *Rainfall (mm)* | *RP (years)* | *Rainfall (mm)* | *RP (years)* |
| *1* | *87.20* | *15* | *81.60* | *10* |
| *5* | *92.00* | *3* | *151.20* | *35* |
| *10* | *108.00* | *2* | *152.40* | *7* |
| *20* | *125.40* | *< 2* | *183.00* | *4* |
| *30* | *192.00* | *3* | *189.20* | *3* |
| *60* | *254.80* | *3* | *246.60* | *3* |
| *90* | *314.00* | *3* | *295.40* | *3* |
| *120* | *378.60* | *4* | *426.20* | *10* |
| *180* | *482.20* | *7* | *525.20* | *15* |

*Table S1: Calculated return period (RP) for the maximum values of rainfall recorded before the start of the landslide period, cumulated in a time range from 1 to 180 days.*
